# Parental traditional medicine use to children and its associated factors at University of Gondar Comprehensive Specialized Hospital: a cross-sectional study

**DOI:** 10.3389/fpubh.2025.1531501

**Published:** 2025-04-28

**Authors:** Masho Tigabe Tekle, Abdisa Gemedi Jara, Mulugeta Endalamaw Ayenew, Banchiamlak Zergaw Toru, Faisel Dula Sema

**Affiliations:** ^1^Department of Clinical Pharmacy, School of Pharmacy, College of Medicine and Health Sciences, University of Gondar, Gondar, Ethiopia; ^2^Department of Pediatric and Child Health, School of Medicine, College of Medicine and Health Sciences, University of Gondar, Gondar, Ethiopia

**Keywords:** traditional, medicine, children, parents, factors, use

## Abstract

**Background:**

The use of traditional medicine (TM) among different group of population has recently shown explosive growth. However, there is limited evidence regarding the extent and its associated factors of using TM for children. This study aimed to determine the prevalence and associated factors of parental TM use for children at University of Gondar Comprehensive Specialized Hospital, Northwest Ethiopia (UOGCSH), 2023.

**Methods:**

A cross-sectional study was conducted among 401 parents who had <15 years old sick children from November 1, 2022 to April 30, 2023. The data was entered into EPI DATA software (4.6.0.2) and analyzed by SPSS version 25. Binary logistic regression was used to identify factors associated with parental TM use and reported with a 95% CI. *p*-value <0.05 was considered statistically significant.

**Results:**

The prevalence of parental TM use for children within the last 12 months was found to be 86.5% (95% CI: 82.50–90.00). Good perception of the parents on level of efficacy of TM (AOR = 3.658; 95%CI: 1.255–10.660), history of using TM for self (AOR = 3.792; 95%CI: 1.783–8.066), occupation; being housewife (AOR = 3.760; 95%CI: 1.515–9.333), daily laborer (AOR = 2.860; 95%CI: 1.035–7.908), and government employee (AOR = 4.134; 95% CI: 1.241–13.778) were significantly associated with parental TM use.

**Conclusion:**

In the present study parental TM use to children was high. Parents who had good perception on level of efficacy of TM, history of taking TM for themselves, housewife, daily laborer and government employee were more likely to use TM for their children. Thus, educating of parents about TM is essential. Further, emphasis should be given for future studies on efficacy and safety of TM use in children.

## 1 Introduction

Traditional medicine (TM) is defined as “health practices, attitudes, knowledge, and beliefs that incorporate manual therapies, spiritual therapies, plant, animal, and mineral-based medications, and methods and exercises used singly or in combination (1). Approximately, 80% of the global population utilizes TM and the use is higher (95%) among developing countries (2). In Africa, approximately 90% of the population depends on TM use, especially in sub-Saharan Africa. In Ethiopia around 80% of the population relies on herbal medicine as their primary source of health care (2, 3).

Despite there was variation in the utilization of TM to children between countries; parental TM use to children was higher with an estimated rate of 30 to 90.3% (4, 5) and its prevalence rate was higher in low income countries. Its high prevalence in developing countries might be explained as the poor economic status of the parents which leads them to frequently use TM for health of their children compared to the modern medicine (4–7). In Ethiopia even though most of previous studies were conducted in adult population, very few studies reported that the prevalence of parental TM use to children is high which reaches up to 90.3% (8–10).

Evidences documented that socio-demographics characteristics of both children’s (i.e., girl, younger age) and parents’ (i.e., female sex, older age, farmer occupation, rural residence, low income, low educational level, and having a good attitude about TM) were associated with high utilization of TM. In addition, TM-related characteristics such as easy accessibility, low price, and effectiveness of TM were factors primarily associated with increased utilization of TM among children (4, 7, 8, 10–15). In Ethiopia, easily accessibility, low price, low educational level, inaccessibility of modern health services, closely corresponding to the patient’s ideology, and less paternalistic than modern medicine leads people to depend on TM for their healthcare needs (1, 9, 15, 16). Other factors such as presence of different biodiversity, beliefs, cultures, and languages among Ethiopian population also contribute to increased of utilization of TM (9, 17). In Ethiopia, studies conducted so far are very limited; focused on adult TM practices (18–21) and the use of TM has not been widely studied among children. In addition, TM in Ethiopia is not uniformly practiced and its uses are considerably diverse and vary significantly between regions (22). The standard policies and regulations to govern TM use are too weak or do not exist at all. Instead, the users are associated with cultural beliefs. Both TM practices and child health are priority issues of Ethiopia. However, little is known about factors for TM use by parents for children in Ethiopia and to the best of the authors’ literature search; no study have been conducted in the study setting. This study was aimed to determine the prevalence and associated factors of parental TM use for children at the pediatric outpatient department ward (POPD) ward of the University of Gondar Comprehensive Specialized Hospital, Northwest Ethiopia (UOGCSH), Northwest Ethiopia.

Even though TM plays an important role among the Ethiopian society, the national health system neither studied the full therapeutic potential of TM nor estimated that the accurate potential adverse effects. In Ethiopia more than half of all TM are prescribed, dispensed as well as sold illegally by a traditional healer and half of all users take TM medicine incorrectly (23). Moreover, as children are dependent on their parents and cannot fight for their health rights, some harmful traditional health practices were found to be killing practices for children. These practices violating the children’s health rights and hindering the country from achieving millennium development goal (MDG 4) (24). Excessive utilization of TM to children is associated with negative or dangerous effects such as developing complications and many children are hospitalized due to adverse effects of TM (25, 26). In addition to this, utilization of TM causes delays in seeking medical care in case of fatal diseases especially in children (7, 27–29).

Since neither the full therapeutic potential nor the potential adverse effects of TM is not well estimated (17), better regulation and control of utilization of TM in vulnerable group of population particularly in children is mandatory. For developing policies which are concerned with better control, regulation and appropriate use of TM understanding the factors associated with increased childhood TM utilization is very crucial and it will fill the knowledge gaps regarding extent of parental TM to children and its determinants. The finding of the study will serve as a baseline information for policy decisions of international organizations which works for safe and effective use of TM and it will assist in supporting the achievement of the WHO TM utilization goal “promoting safe and effective use of TM through regulation, evaluation, and integration of TM products, practices and practitioners into health systems as appropriate” (22) and giving direction to minimize the deleterious effect of excessive utilization of TM on the lives of children. Further, this study will provide baseline information for similar studies that are going to be conducted in the future.

## 2 Methods

### 2.1 Study design, area, and period

A cross-sectional study was conducted at the POPD ward of UOGCSH from November 1, 2022 to April 30, 2023 which is found in Gondar town, 738 km away from the capital city, Addis Ababa. Currently, the hospital serves as a teaching and a referral hospital to other governmental hospitals, health centers, and private clinics from surrounding areas. Pediatric Out–Patient Department (POPD) is a department where sick children are evaluated, treated, and monitored. Currently, at UOGCSH, on average, 45 children/day = 1,350/month visit the POPD.

### 2.2 Population inclusion and exclusion criteria

All parents who had <15 years old sick children and attended the POPD of UOGCSH were the source population. Parents who had <15 years old sick children, live with their children for ≥1 year, attended the POPD of UOGCSH and were willing to participate in the study during the study period were included as the study population. Whereas, parents who had a seriously sick child or were unable to give the required information during the data collection period were excluded.

### 2.3 Sample size determination and sampling procedure

The minimum number of samples required for the study was determined by using single population proportion formula considering the following assumptions:


$$n_{0}=\frac{Z^{2}p\left(1-P\right)}{d^{2}}$$

$$n_{0}=\frac{{\left(1.96\right)}^{2}*0.5*\left(1-0.5\right)}{{\left(0.05\right)}^{2}}$$

$$n_{0}=384$$

Where: n_0_ = minimum sample size required for the study; Z = standard normal distribution (Z = 1.96) with a CI of 95% and ⍺ = 0.05; P = expected prevalence of parental TM use for children (*p* = 50%); W = Absolute precision (W) = 0.05. Then, adding 10% (384 × 0.1 = 38.4 ≈ 39) of the non-respondent rate, the total sample size for the study was (384 + 39) = 423. Parents who had under fifteen years of age sick children (study participants) of the study were selected using a systematic random sampling technique. Since the sample size of the study was decided to be 423, the sampling fraction (the K value) was obtained by dividing the total number of children having service in 1 month (1350) for the total sample size (423) (1,350/423 = 3.191 ≈ 3). Thus, parents were selected at regular intervals of 3 (i.e., every 3rd) while the 1st parent was selected by lottery method.

### 2.4 Study variables

The dependent variable was parental TM use for children within the last 12 months and it was described as frequency (percentage). Independent variables included were the socio-demographic characteristics of children (sex, age, and breastfeeding status), socio-demographic characteristics of parents (sex, age, number of children in a family, religion, marital status, occupation, education, and residence), enabling and need factors (sources of information about TM a child is using, reasons for using TM to children, and type of a disease for which TM is used for a child), health care experience (history of parental TM use for themselves, parental level of satisfaction after using TM for themselves, perception of parents on the level of efficacy of TM, reasons for using TM than modern medicine for themselves, TM disclosure to physicians/ nurse and reasons for non-disclosure), TM related characteristics (type, indication, route of administration, and source of TM a child is using).

### 2.5 Data collection instrument and procedures

Data was collected using a pretested structured interviewer-administered questionnaire by three trained pharmacists and one supervisor. The mother or the father or the guardian of the children was interviewed. But priority was given for the mother because mothers are close to their children than fathers. When the mothers were not available by any means, the father or the guardian who were living with the child for the past 12 or more consecutive months were interviewed. The questionnaire was developed by adapting from similar studies conducted previously (8–10, 15) and the first part of the questionnaire consisted of socio-demographic variables which show children's and parents’ background information. The second part comprised questions used to assess the prevalence of TM use practice for children. Additionally, this part contains variables used to collect information about TM-related characteristics such as type, source, and route of administration. The third part included questions that address the enabling and need factors. The fourth part contained questions related to the healthcare experience of the parents. The fifth part of the questionnaire includes questions regarding TM disclosure to physicians/nurses and reasons for non-disclosure (Supplementary material).

### 2.6 Data quality control technique

The questionnaire was prepared in English and translated to the local language Amharic and back translated to English. To check the consistency of the questionnaire, 2 weeks before the actual data collection, a pretest was carried out among 45 parents (10% of the total sample size) at POPD of Debra Tabor Hospital and modification was done on the questionnaire accordingly. In addition, the data collected for the pretest was excluded from analysis. Before data collection, training was given to the three pharmacists and one supervisor concerning the objective and the process of data collection. Additionally, during the data collection period, supervision was held regularly and the collected data were checked daily for completeness and consistency.

### 2.7 Data entry and statistical analysis

Data were cleaned, coded, entered into EPI DATA version 4.6.0.2, and exported to Statistical Package for Social Sciences (SPSS) version 25 for analysis. Variables related to the socio-demographic characteristics of both children and parents, the prevalence of TM use, TM-related characteristics, enabling and need factors, health care experience of the parents were described using descriptive statistics. Kolmogorov–Smirnov test was used to test data normality (*p* > 0.05 = normal distribution). Since the continuous variables were not normally distributed, they were expressed as median (IQR). Additionally, categorical variables were summarized as frequency (percentage) of the total. Multicollinearity was checked using Variance Inflation Factor (VIF). Multivariable binary logistic regression was used to identify factors associated with the prevalence of parental TM use to children. Both crude odds ratio (COR) and adjusted odds ratio (AOR) with the corresponding 95% confidence interval (CI) were used to measure the strength of association. A *p*-value <0.05 in the multi-variable regression model was considered statistically significant. The fitness of the logistic regression model was evaluated by the Hosmer–Lomeshow test (Chi-square (χ2) = 3.144, *p*-value = 0.925).

### 2.8 Operational definition

Children: person under the age of 15 years old.
Parent: father, mother, or/and guardian who raises a child.

Traditional medicine: defined as “health practices, attitudes, knowledge, and beliefs that incorporate manual therapies, spiritual therapies, plant, animal, and mineral-based medications, and methods and exercises used singly or in combination Traditional medicine utilization: using of anything used in the promotion of health, prevention of illness, and treatment of diseases and is not currently considered to be part of modern medicine but accepted in that community. TM is neither prescribed by the healthcare professional nor used commonly as a diet in that particular culture.

Parental TM use for children: using of TM for a child by parents which was described as the frequency (percentage) of parents who have ever used TM for their children within the last 12 months.

### 2.9 Ethics approval and consent to participate

The study was conducted in accordance with the ethical principles of the declaration of Helsinki. The ethical approval was obtained from the Ethical Review Committee of School of Pharmacy College of Medicine and Health Sciences, University of Gondar with ethical approval number SOP/257/2022. Before data collection the purpose of the study was clarified to each parent and written informed consent was obtained from each parent who was involved in the study. Among the parents who had ≥10 years old child, in addition to the written informed consent, by providing description about the purpose of the study oral assent was obtained from their child.

## 3 Results

### 3.1 Socio-demographic characteristics of children and parents

In the study, a total of 401 parents with a corresponding response rate of 94.79% were included.

The median (IQR) age of children and parents was 5 (IQR: 3–9) and 32 (IQR: 28–39) years, respectively. The majority (79.6%) of the parents were females (mothers: 77.8%), and (69.3%) of the respondents were living in urban areas. More than half (58.6%) of the parents were member of community-based health insurance. Nearly half (51.9%) of the children were boys and less than a quarter (20.7%) of them were breastfeeding (Table 1).

**Table 1:** Socio-demographic characteristics of parents at UOGCSH, Northwest, Ethiopia, 2023 (*N* = 401).

Variable	Category	Frequency (%)
Age	18–29 years	126 (31.4)
	30–44 years	209 (52.1)
	45–59 years	55 (13.7)
	≥ 60 years	11 (2.7)
Sex	Male	82 (20.4)
	Female	319 (79.6)
Marital status	Single	18 (4.5)
	Married	355 (88.5)
	Widowed	9 (2.2)
	Divorced	19 (4.7)
Level of education	Not able to read and write	99 (24.7)
	Able to read and write	21 (5.2)
	Primary	76 (19.0)
	Secondary	96 (24.0)
	College and above	109 (27.2)
Occupation	Housewife	149 (31.9)
	Farmer	52 (11.0)
	Merchant	29 (6.2)
	Daily laborer	79 (16.9)
	Government employee	55 (11.8)
	Private organization employee	37 (7.9)

%; percent; UOGCSH, University of Gondar Compressive Specialized Hospital.

### 3.2 Prevalence of parental TM use for their children, enabling and need factors for parental TM use

**Figure 1:**
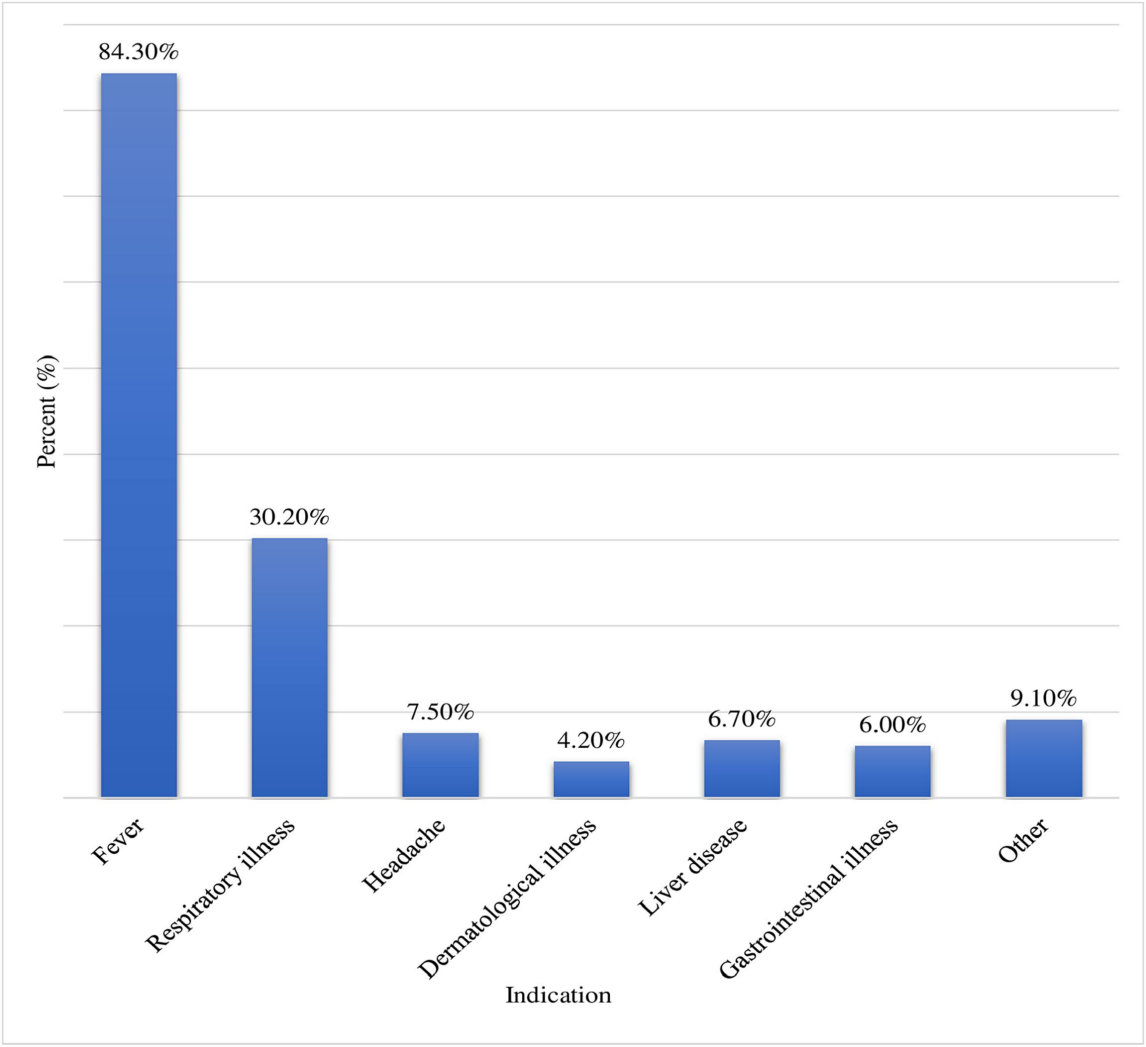
Indications for TM use for children at UOGCSH, Northwest, Ethiopia, 2023 (*N* = 401). Other: animal bite, ear problem, deworming, wound, convulsion, evil eye, lethargy; GI: gastrointestinal.

**Figure 2:**
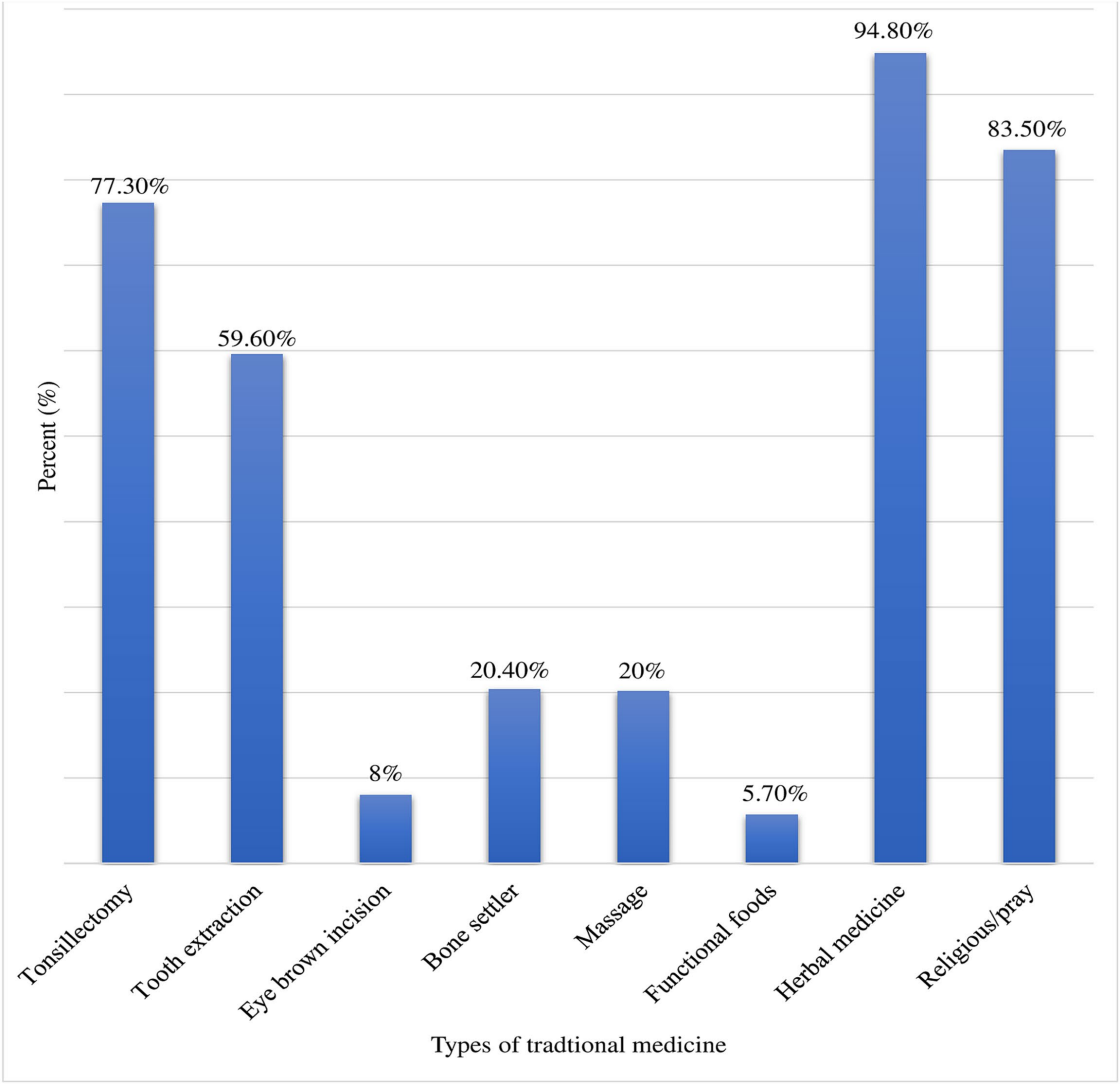
Types of TM used by children at UOGCSH, Northwest, Ethiopia, 2023 (*N* = 401).

The prevalence of parental TM for their children within the last 12 months was 86.5% (95% CI: 82.5–90.00). Moreover, a significant number (86.8%) of the parents mentioned that they had a plan for using TM in the future for their children and they encourage other persons to use TM for children (70.1%). Cultural belief (36.9%) which was followed by religious belief (28.69%) and easy accessibility (17.41%) was major reason to use TM for children. Family (60.17%) and neighbors (34.26%) were the two most frequent sources of information for parents to use TM for their children (Table 2). Majority of the parents (72.6%) were using TM to treat their children’s illnesses; fever (84.3%) followed by respiratory illness (30.2%) was the most frequent illness for which TM was indicated (Figure 1). Herbal medicine (94.8%), religious/prayer (83.5%), tonsillectomy (77.3%), and tooth extraction (59.6%) were the most commonly used types of TM for children (Figure 2).

**Table 2:** Prevalence of parental TM use for children, enabling and need factors for parental TM use at UOGCSH, Northwest, Ethiopia, 2023 (*N* = 401).

Variable	Category	Frequency (%)
Ever use of TM for children (*N* = 401)		
	Yes	359 (89.5)
	No	42 (10.5)
Parental TM use for their children with in the last 12 months (*N* = 401)		
	Yes	347 (86.5)
	No	54 (13.5)
When have you used TM for your child in recent time? (*N* = 359)		
	Within 1 month	64 (17.83)
	Before a month but within 6 months	133 (37.05)
	Before 6 months	162 (45.12)
Status of a child after using TM (*N* = 359)		
	Very poor	43 (12.0)
	Poor	102 (28.4)
	Fair	81 (22.6)
	Good	84 (23.4)
	Very good	49 (13.6)
Route of administration (*N* = 359)		
	Inhalational	136 (37.88)
	Topical	106 (29.53)
	Oral	98 (27.29)
	Other*	19 (5.30)
Reasons for using TM to children (*N* = 359)		
	Cultural belief	148 (36.9)
	Religious belief	103 (28.69)
	Being referred by someone	33 (9.20)
	Other**	28 (7.80)
Sources of information about TM a child is using (*N* = 359)		
	Family	216 (60.17)
	Neighborhoods	123 (34.26)
	Religious institutions	11 (3.06)
	Other***	9 (2.51)
Source of TM (*N* = 359)		
	Cultivated	191 (53.20)
	Wild	98 (27.3)
	Prepared at home	41 (11.42)
	From traditional healer	29 (8.08)
For which type of a disease you have used TM for your child (*N* = 359)		
	For a disease which lasts <1 week	262 (72.98)
	For a disease which lasts 1–2 week	56 (15.60)
	For a disease which lasts 3–4 week	27 (7.52)
	For a disease which lasts >4 week	14 (3.90)
Do you have a plan for using TM for your children in the future? (*N* = 401)		
	Yes	348 (86.8)
	No	53 (13.2)
Do you encourage other persons to use TM for children? (*N* = 401)		
	Yes	281 (70.1)
	No	120 (29.9)

% = percent; TM, traditional medicine; UOGCSH, University of Gondar Compressive Specialized Hospital; Other*: kitab, bathing; Other**: family influence, when there is no improvement with modern medicine; Other***: friends, television/internet/media, traditional healer.

### 3.3 Health care experience of the parents

More than two-thirds (88.5%) of the participants stated that they have used any form of TM within the previous 12 months for themselves. Believing that some diseases are not cured by modern medicine (40.0%) and when selected correctly it is effective (27.61%) were the main reasons for using TM more than modern medicine (Table 3).

**Table 3:** Health care experience of the parents at UOGCSH, Northwest, Ethiopia, 2023 (*N* = 401).

Variable	Category	Frequency (%)
Have you used any form of TM use for yourself with in the last 12 months (*N* = 401)		
	Yes	355 (88.5)
	No	46 (11.5)
What was your level of satisfaction after using TM for yourself? (*N* = 355)		
	Completely satisfied	70 (19.7)
	Somewhat satisfied	88 (24.8)
	Neutral	58 (16.3)
	Somewhat dissatisfied	78 (22.0)
	Completely dissatisfied	61 (17.2)
What is your perception on the level of efficacy of TM? (*N* = 401)		
	Very poor	35 (8.7)
	Poor	49 (12.2)
	Fair	62 (15.5)
	Good	110 (27.4)
	Very good	145 (36.2)
What was your reason for using TM than modern medicine for your-self (*N* = 355)?		
	Believing there are some diseases which are not curb by modern medicine	142 (40.0)
	When selected correctly it is effective	98 (27.61)
	Satisfaction with traditional medicine	51 (14.36)
	Cheap compared to modern medicine	32 (9.01)
	The fear of using drugs and their side effects	16 (4.51)
	Dissatisfaction with modern medicine	10 (2.82)
	Having good knowledge about traditional medicine	15 (3.2)
	Believing less efficacy of modern medicine	6 (1.69)

% = percent; TM, traditional medicine.

### 3.4 Traditional medicine use disclosure to healthcare professionals

Regarding the disclosure of TM, most of the parents (87.3%) had not disclosed TM use to healthcare professionals and only (12.7%) reported they had disclosed TM use for their children. The majority of the participants (88.7%) explained that not being asked by a healthcare professional was their primary reason for not disclosing TM. However, a significantly higher number of respondents stated that they would discontinue TM if their children were getting sicker after giving TM (94.5%) and if their doctor asked them to stop TM (80.8%).

### 3.5 Factors associated with parental traditional medicine use for children

Having good perception of the parents regarding the level of efficacy of TM, history of using TM for self, occupation specifically; being housewife, daily laborer, and government employee were significantly associated with parental TM use for children. Compared to parents who had a very poor perception about the level of efficacy of TM, those who had good perception were 3.658 times more likely to use TM for their children (AOR = 3.658; 95%CI: 1.255–10.660). Parents who take TM for themselves were 3.792 times more likely to use TM for their children compared to their counterparts (AOR = 3.792; 95%CI: 1.783–8.066). Additionally, parents who were house wife (AOR = 3.760; 95%CI: 1.515–9.333), daily laborer (AOR = 2.860; 95%CI: 1.035–7.908), and government employee (AOR = 4.134; 95%CI: 1.241–13.778) were 3.760, 2.860, and 4.134 times more likely to use TM for their children when they compared with parents who were employed in private organization, respectively (Table 4).

**Table 4:** Factors associated with parental traditional medicine used for children at UOGCSH, Northwest, Ethiopia, 2023 (*N* = 401).

Variables	Category	Parental TM use		COR (95%CI)	AOR (95%CI)	*p*-value
		Yes	No			
Occupation						0.080
	Housewife	132	17	3.727 (1.587, 8.753)	3.760 (1.515, 9.333)	0.004*
	Farmer	47	5	4.512 (1.428, 14.258)	3.380 (0.999, 11.433)	0.050
	Merchant	24	5	2.304 (0.705, 7.529)	1.892 (0.545, 6.572)	0.316
	Daily laborer	69	10	3.312 (1.273, 8.614)	2.860 (1.035, 7.908)	0.043*
	Government employee	50	5	4.800 (1.522, 15.136)	4.134 (1.241, 13.778)	0.021*
	Private organization employee	25	12	Ref	Ref	
Cultural belief-reason for using TM for children						
	Yes	135	13	0.498 (0.257, 0.963)	1.585 (0.780, 3.221)	0.203
	No	212	41	Ref	Ref	
Perception on the level of efficacy of TM-health care experience						0.126
	Very good	129	24	2.067 (0.884, 4.835)	1.859 (0.754, 4.581)	0.178
	Good	101	9	4.316 (1.590, 11.714)	3.658 (1.255, 10.660)	0.017*
	Fair	54	6	3.462 (1.135, 10.556)	2.953 (0.920, 9.482)	0.069
	Poor	37	5	2.846 (0.870, 9.308)	3.185 (0.884, 11.472)	0.076
	Very poor	26	10	Ref	Ref	
History of using TM for self						
	Yes	316	39	0.255 (0.127, 0.514)	3.792 (1.783, 8.066)	0.001^*^
	No	31	15	Ref	Ref	

^*^ Statistically significant at *p* < 0.05; Ref, reference category; COR, crude odd ratio; AOR, adjusted odd ratio; CI, confidence interval; TM, traditional medicine.

## 4 Discussion

Despite there is explosive growth in the utilization of TM among different group of population, neither the full therapeutic potential nor the potential adverse effects of TM is not well estimated (17). Thus, ensuring safe, effective and appropriate utilization of TM through better regulation and control of utilization of TM in vulnerable group of population particularly in children is mandatory. For achieving this, understanding the factors associated with increased childhood TM utilization is very crucial. Thus, purpose of this study was to determine the prevalence and associated factors of parental TM use for children at the pediatric outpatient department ward (POPD) ward of the UOGCSH, Northwest Ethiopia. In the current study, the prevalence of parental TM use for children within the last 12 months was 86.5% (95% CI: 82.50–90.00). Having good perception of the parents regarding the level of efficacy of TM, history of using TM for self, occupation specifically; being housewife, daily laborer, and government employee were significantly associated with parental TM use for children.

This study showed that parental TM use to children was 86.5% (95% CI: 82.50–90.00). This finding was almost consistent with the previously conducted studies in Ethiopia, Motta town (88.2%) (12), Tole District (85.9%) (17), and Mecha District (90.3%) (13). However, it was higher than studies done in Korea (65.3%) (18), Wales (41%) (19), Sudan (70%), Nigeria, (29.5%) and Southern Arizona (64%) (20). The reason for this high prevalence might be Ethiopia’s low socio-economic status in which 90% of its population to relied on TM (30). In addition to the poor economic status of the parents which leads them to use TM more frequently to their children than modern medicine, the presence of different biodiversity, beliefs, cultures, and languages among Ethiopian population may also contribute to increased of utilization of TM to children (9, 17, 27, 29). Further possible explanation for this difference might arise from methodological and cultural differences between the studies area. In addition, the use of TM for their children within the previous 1 month was 17.83%, which was similar to a study conducted in Motta (19.2%) (12) and Fagita Lekoma (17.3%), Ethiopia (11). This result was higher than a research done in Indonesia (6.2%) (16) and Taiwan (4.7%) (15).

In this study the most frequent illness for which TM was indicated were fever (84.3%) and respiratory illness (30.2%). This was in line with a study conducted in Turkey (8). This might be explained by the high prevalence of respiratory infectious disease specifically community-acquired pneumonia in the study setting of the current study (21).

Herbal medicine (94.8%) and religious/pray (83.5%) were the most commonly used types of TM for children. This was supported by previous studies which were conducted to determine the prevalence of caregivers’ utilization of TM for their children (8, 9, 12, 20, 22). An interesting finding in this study was tonsillectomy (77.3%) and tooth extraction (59.6%) were among the most commonly used types of TM. This is finding implies that healthcare providers need to educate parents about the disadvantages of home based tonsillectomy and tooth extraction procedures with the help of traditional healer, as these procedures may put children at high risk for future cardiac illness such as acute rheumatic fever and infective endocarditis.

The parents described that cultural belief (36.9%), religious belief (28.69%), and easy accessibility (17.41%) were their main reasons to use TM for children. Other studies also reported that cultural belief, religious belief, and easy accessibility were the major reasons for using TM for children (11, 23). Literatures also described that, in Ethiopia, as the users are associated with cultural beliefs, TM is not uniformly practiced and its uses are considerably diverse and vary significantly between regions (25, 26). In the present study family (60.17%) and neighbors (34.26%) were the two most frequent sources of information for the parents to use TM for their children. Supporting this finding several studies described that both family and neighbors were the most common sources of information for using TM in children (8, 11, 12, 24).

In this study, most of the parents (87.3%) reported that they did not disclose TM use and not being asked by the health care professional (88.7%) about their use was their primary reason for not disclosing. This was in agreement with previous studies, which showed that 49 to 92% of the parents had not disclosed complementary and alternative medicine use in their children to a physician/nurse (19, 24–26). A significantly higher number (80.8%) of respondents stated that they would discontinue TM if their doctor asked them to stop TM. Similarly, a study in Ghana also found that more than three-fourths (88%) of households said they would withhold herbal medicines if a child was taking modern medicines (27). This finding highlight that, during provision of healthcare services to children, healthcare professionals should ask parents regarding whether they give any TM including herbal medicine to their child not.

In this study compared to parents who had a very poor perception about the level of efficacy of TM, those who had good perception were 3.658 times more likely to use TM for their children. Inconsistent with this finding, previous studies demonstrated that positive attitude, favorable perception and stronger belief about effectiveness of TM and CAM was significantly associated with using TM and CAM (12, 28, 29, 31). In the present study parents who had history of taking TM for themselves were 3.259 times more likely to use TM for their children compared to those who had no history of taking TM. A previous study in Ethiopia also described that parental self-TM use was significantly associated with higher utilization of TM for children (32). Similarly, a study conducted in Iran also explained that parents with previous positive experiences of CAM were significantly more likely to use CAM for their children (30). In this study, parents who were house wife, daily laborer, and government employee were 3.760, 2.860, and 4.134 times more likely to use TM for their children when they compared with parents who were employed in private organization, respectively. This result was comparable with the study conducted in Southwest Ethiopia which indicated that house wife and government employee were more likely to practice TM compared to other group of occupations (33). In Ethiopia, because of its easy accessibility and affordability, TM is used as an alternative medical practice. However, its excess utilization in pediatric age group of population may leads to some adverse clinical effects. Further, despite both TM practices and child health are priority issues of Ethiopia. The standard policies and regulations to govern TM use are too weak or do not exist at all. Thus, assessment of the major reasons for high utilization of TM to children is important for developing strategies which are essential for better control and regulation of TM utilization in children. Thus, finding of this study will fill the information gap regarding the magnitude and factors associated with utilization of TM children specifically in the study setting. Educating of parents about TM is essential and emphasis should be given for future studies on efficacy and safety of TM use in children. Since the current study was conducted cross-section ally, cause-effect relation between practice of parental TM use for children and its significant associated factors cannot be established. As it assessed past history of utilization of TM it experience recall bias. Additionally, it was carried out in a single centered health facility and this may limit the generalizability of the study findings.

## 5 Conclusion

This study showed that the prevalence of parental TM use to children was high. Parents who had good perception on level of efficacy of TM, history of taking TM for themselves, housewife, daily laborer and government employee were more likely to use TM for their children. This study highlight parent related characteristics specifically past history of using TM, occupation; housewife, daily laborer and government employee, and their positive perception towards TM efficacy were factors associated with increased use of TM to children. Thus, educating of parents about TM is essential. Further, emphasis should be given for future studies on efficacy and safety of TM use in children.

## Data Availability

The original contributions presented in the study are included in the article/Supplementary material, further inquiries can be directed to the corresponding author.
