# Supplementary material for: Parental traditional medicine use to children and its associated factors at University of Gondar Comprehensive Specialized Hospital: a cross-sectional study
Source: Front Public Health. 2025 Apr 28;13:1531501. doi: 10.3389/fpubh.2025.1531501 (PMC12066296; doi:10.3389/fpubh.2025.1531501)
Supplement: Supplementary file 1 [file Data_Sheet_1.pdf]

Supplementary Material: Questionnaire

Part I: Socio-demographic characteristics of children and parent

Instruction: Please circle the number in front of the option you choose

No.	Variable	Coding categories
101	Sex of the child	1. Male 0. Female
102	Age of the child	___________ (years)
103	Breast feeding status	1. Yes 0. No
104	Sex of the parent	1. Male 0. Female
105	Age of the parent	___________ (years)
106	Relationship with the child	1. Mother<br>2. Father<br>3. Brother<br>4. Sister<br>5. Other
107	Marital status	1. Married<br>2. Single<br>3. Widowed<br>4. Divorced
108	Educational status of the parents	1. Notable to read and write<br>2. Able to read and write<br>3. Primary school (1-8)<br>4. High school (9-12)<br>5. Above high school (>12)
109	Occupation of the parents	1. Housewife<br>2. Farmer<br>3. Merchant

		4. Daily laborer<br>5. Governmental employed<br>6. Private organization employed
110	How many children do you have (up to 15 years)?	______________
111	Are you a member of community-based health insurance?	1. Yes 0. No
112	Residence	1. Urban 0. Rural

Part II: Traditional medicine use practice to children

Instruction: Please circle the number in front of the option you choose

No.	Variable No	Coding categories
201	Have you ever used any form of TM for your child (yes/no)?	1. Yes 0. No
202	Have you used any form of TM for your child with in the last 12 months (yes/no)?	1. Yes 0. No
203	When have you used TM for your child in recent time?	1. Within 1 months<br>2. Before a month but within 6 months<br>3. Before 6 months
204	For what purpose you used Traditional Medicine for your child (you can choose more than one option)?	1. To promote health<br>2. To prevent illness<br>3. To treat illness/symptom relief<br>4. Others (specify)………
205	What was the status of your child after using TM?	5. Very poor<br>6. Poor<br>7. Fair<br>8. Good<br>9. Very good
206	What types of traditional medicine you used for your children (you can choose more than one option)?	1. Religious/pray/Tsebel<br>2. Tonsillectomy<br>3. Teeth extraction

		4. Eye brown incision<br>5. Bone settler<br>6. Message<br>7. Functional foods<br>8. Herbal medicine (ginger, garlic, demacase, etc)<br>9. Any others (specify) _________
207	What are the routes of administration of the traditional medicine you used for your child (you can choose more than one option)?	1. Orally<br>2. Bathing<br>3. Dermal<br>4. Inhalational<br>5. Any other (specify) ___________
208	What was your source of traditional medicine while you were using to your child/form where you get (you can choose more than one option)?	1. Cultivated<br>2. Wild<br>3. From traditional healer<br>4. Prepared at home
213	Do you have a plan for using traditional medicine for your children in the future (yes/no)?	1. Yes 0. No
214	Do you encourage other persons to use traditional medicine for children (yes/no)?	1. Yes 0. No

Part III: Enabling and need factors for parental traditional medicine use to children

Instruction: Please circle the number in front of the option you choose.

No.	Variable	Coding categories
301	What are your sources of information about the traditional medicine for your child is using? (You can choose more than one option)?	1. Family/relative<br>2. Neighborhoods<br>3. Religious institutions<br>4. Friends<br>5. Television/internet/media,<br>6. Traditional healer

302	For which child's symptom you used traditional medicine (you can choose more than one option)?	1. Crying<br>2. To increase immunity<br>3. Evil eye disease<br>4. Fever<br>5. Liver disease<br>6. Gastrointestinal disease (nausea vomiting, diarrhea)<br>7. Headache<br>8. Teething<br>9. Dermatological problem (rash)<br>10. Respiratory illness (cough, fast and noisy breathing)<br>11. Any others? (Specify) ___________
303	For which type of a disease you have used TM for your child	1. For a disease which lasts < 1 week<br>2. For a disease which lasts 2-3 week<br>3. For a disease which lasts 3-4 week<br>4. For a disease which lasts > 4 week
304	What is your reasons for using Traditional Medicine?	1. Religious belief<br>2. Family influence<br>3. Cultural belief<br>4. Referred by someone<br>5. Being easily accessible<br>6. When there is no improvement with modern medicine<br>7. Any others? (specify) ______

Part IV: Health care experience of the parents

Instruction: Please circle the number in front of the option you choose.

No.	Variable	Coding categories
401	Have you used any form of TM use for yourself with in the last 12 months (yes/no)?	1. Yes 0. No
402	What was your level of satisfaction after using TM for yourself?	1. Completely satisfied<br>2. Somewhat satisfied

		3. Neutral<br>4. Somewhat dissatisfied<br>5. Completely dissatisfied
403	What is your perception on the level of efficacy of TM?	1. Very poor<br>2. Poor<br>3. Fair<br>4. Good<br>5. Very good
404	What was your reason for using TM than modern medicine for your-self?	1. Believing there are some diseases which are not curb by modern medicine<br>2. When selected correctly it is effective<br>3. Satisfaction with Traditional Medicine<br>4. Cheap compared to modern medicine<br>5. The fear of drugs side effects<br>6. Dissatisfaction with modern medicine<br>7. Believing less efficacy of modern medicine<br>8. Having good knowledge about traditional medicine<br>9. Any others? (specify)-------

Part V: Disclosure regarding practice of TM use to children

Instruction: Please circle the number in front of the option you choose.

**No**	**Variable**	**Coding categories**
501	If you used TM for your child, and a doctor or nurse asked you about the TM would you disclose (yes/no)?	1. Yes 0. No
502	If you say no for question no "209" what was your reason for not disclosing (you can choose more than one option)?	1. Not being asked by a healthcare professional<br>2. The staff will not understand

		3. The fear of the staff<br>4. It is not relevant to tell
503	If your Doctor/nurse asked you to stop giving traditional medicine to children, would you stop?	1. Yes 0. No
504	If your child getting sicker after giving traditional medicine would you stop to give?	1. Yes 0. No

Thank you for your time!!!
